# Atypical connectivity aids conversation in autism

**DOI:** 10.1038/s41598-023-32249-5

**Published:** 2023-03-31

**Authors:** Kyle Jasmin, Alex Martin, Stephen J. Gotts

**Affiliations:** 1 Section on Cognitive Neuropsychology, Laboratory of Brain and Cognition, National Institute of Mental Health, NIH, Bethesda, MD USA; 2 Department of Psychology, Royal Holloway, University of London, Egham, Surrey UK

**Keywords:** Neuroscience, Cognitive neuroscience, Language, Autism spectrum disorders

## Abstract

It is well-established that individuals with autism exhibit atypical functional brain connectivity. However, the role this plays in naturalistic social settings has remained unclear. Atypical patterns may reflect core deficits or may instead compensate for deficits and promote adaptive behavior. Distinguishing these possibilities requires measuring the ‘typicality’ of spontaneous behavior and determining how connectivity relates to it. Thirty-nine male participants (19 autism, 20 typically-developed) engaged in 115 spontaneous conversations with an experimenter during fMRI scanning. A classifier algorithm was trained to distinguish participants by diagnosis based on 81 semantic, affective and linguistic dimensions derived from their use of language. The algorithm’s graded likelihood of a participant's group membership (autism vs. typically-developed) was used as a measure of task performance and compared with functional connectivity levels. The algorithm accurately classified participants and its scores correlated with clinician-observed autism signs (ADOS-2). In support of a compensatory role, greater functional connectivity between right inferior frontal cortex and left-hemisphere social communication regions correlated with more typical language behavior, but only for the autism group. We conclude that right inferior frontal functional connectivity increases in autism during communication reflect a neural compensation strategy that can be quantified and tested even without an a priori behavioral standard.

## Introduction

Neural studies of autism have often used functional connectivity analyses to identify networks that are under- or over-connected relative to neurotypical controls. Many of these studies have assessed connectivity during the resting state, and have identified both decreases (often among cortical social processing areas), and increases (often involving subcortical structures)^1–3^, although much heterogeneity clearly exists^4^. Other studies have examined connectivity during social or language tasks, which also report both decreases^5,6^ and increases^7^ in connectivity. However, the relationship between atypical connectivity and behavior has remained unclear. Some atypical patterns may cause social impairments (see^8^). Others, especially those that emerge only during tasks, might instead reflect alternative neural strategies that compensate for core deficits^9,10^. Determining which patterns promote adaptive behavior, and which hinder it will be crucial for understanding autism more fully and developing connectivity-based interventions^8,11^.

One common technique for assessing the role of a given network in behavior has been to test for correlations with some measure of symptom severity, such as the Social Responsiveness Scale^12^, a questionnaire undertaken by parents or teacher that measures aspects of social cognition and autistic mannerisms. This tool has strong psychometric properties and may therefore accurately measure core autism-related deficits, as it is completed by someone (often a parent or teacher) who knows the individual well. However, tools like SRS that measure autistic traits may be insufficient for assessing behavioral compensation. This is because compensation reflects not the severity of core social deficits, but the degree to which overt behavior superficially resembles typical behavior in the face of these core deficits^9^. Assessing neural compensation in complex spontaneous social behavior therefore requires quantifying how typical behavior is during social interactions and then relating the degree of typicality to functional connectivity obtained during the same interactions. Evidence for compensation in autistic participants would consist of increasingly dissimilar functional connectivity relative to neurotypical controls in a relevant brain network as behavior becomes increasingly control-like.

We recently reported a study investigating face-to-face conversation in autism which makes it possible to examine compensatory behavior. Autism and typical participants engaged in spontaneous ‘face-to-face’ social interactions with an experimenter through video and audio links while being scanned with functional MRI^13^. Measurements of neural activity during the task were compared with resting state. Some task and resting state results were similar, namely increased connectivity between subcortical structures (thalamus and ventral striatum) and the cortex. By contrast, the cortico-cortical pattern differed markedly between the resting and task states: during social interaction, widespread increases in connectivity, rather than decreases, were detected within a distributed, bilateral network of brain regions^13^. We hypothesized that the overconnectivity may reflect a compensatory strategy to meet the demands of the difficult social task, but we lacked an objective measure of task performance that would have allowed us to test this account.

Here we now report an objective measure of the autism phenotype as it relates to language produced during the conversations in that experiment, which was developed by training a classifier algorithm on the transcribed speech of the autism and typical participants. The metric was validated by assessing how accurately it classified participants by diagnostic category and how well it correlated with clinician ratings on the ADOS-2, the ‘gold standard’ autism diagnostic interview tool^14^. Finally, functional connectivity was assessed. If connectivity differences during conversation are compensatory, then these differences should correspond to a less pronounced autism phenotype during the interactions.

## Methods and materials

### Participants

Nineteen males (aged 14.7 to 28.2 years) with autism and 20 male typically-developed participants (aged 15.1 to 32.0 years) took part. Participants with autism were recruited from the Washington, DC metropolitan area and met DSM-5 criteria for Autism Spectrum Disorder (APA, 2013) as assessed by an experienced clinician. All participants with autism received the ADOS-2 Module 4^14^. The scores from participants with autism met cut-off for the ‘broad autism spectrum disorders’ category according to criteria established by the National Institute of Child Health and Human Development/National Institute on Deafness and Other Communication Disorders Collaborative Programs for Excellence in Autism^15.^ The distributions for full-scale IQ, verbal IQ, and age did not differ statistically between the autism and typical groups^13^. The experiment was approved by the NIMH Institutional Review Board (protocol 10-M-0027, clinical trials number NCT01031407). All methods were performed in accordance with the relevant guidelines and regulations.

### Procedure

Each session consisted of three spontaneous conversations between the participant and the experimenter. Prior to scanning, participants were told that they would engage in three unstructured and informal conversations. Using a questionnaire^16^, participants rated their level of interest in various topics such as music, games, and transportation vehicles, and indicated their top three interests, from which the experimenter selected two to serve as conversation topics. The topic of the final conversation was always work or school life, depending on participant’s age. The topics of conversations are listed in Supplementary Table 2 of Jasmin et al., 2019^13^. Before each conversation run, the experimenter sat in front of a blue screen facing a camera. The run began with 16 s of rest. Then, live video and audio from the experimenter were presented to the subject and the interaction task began. Conversations proceeded for 6 min. After each interaction, the video faded to black and a ‘STOP’ slide was displayed to the participant, followed by 30 additional seconds of rest to allow for delayed hemodynamic effects.

### Behavioral data processing

Although 117 conversations took place, for two conversations audio recording was unsuccessful and therefore transcription was not possible. These two conversations, from two different typically-developed participants, were excluded from analysis, along with their brain data, resulting in 115 conversations available for analysis. The audio recordings of the conversations were transcribed by professional transcriptionists and the text was analyzed with the Linguistic Inquiry Word Count (LIWC; pronounced “Luke”), 2007 Edition^17^. LIWC outputs 81 variables that reflect linguistic aspects of a text (e.g. total word count, number of words per sentence), as well as counts of words in particular linguistic, semantic and affective categories (e.g. affective words, sensory words, pronouns, articles). LIWC has been used previously to analyze typed language production in autism^18–20^, but not transcribed spoken language as far as we are aware.

The values of the LIWC output variables were z-scored, and a cross-validated linear support vector machine (SVM) model was trained on the conversations using a leave-one-out approach (*CVSVMModel* function in MATLAB 2020b). That is, each conversation was tested with the other 114 conversations serving as the training set. The output scores of the left-out conversations were transformed using the *fitSVMPosterior* function to probabilities that reflected the certainty of the classification decision for each conversation^21^. Lower scores indicated more certainty of autism classification according to the model, and higher scores indicated more certainty of typical classification. The probability scores for each conversation were then averaged together to yield a single composite score for each participant, which if lower than 0.5 indicated an overall classification of autism by the algorithm.

Performance of the classifier was assessed by computing accuracy (percent correct classifications relative to actual diagnosis). Statistical significance of classifier accuracy was determined by permutation test: a null distribution was compiled by permuting the diagnosis label for the participants, running a leave-one-out cross-validation (as above, with one conversation left out), and averaging the resulting probability scores by participant. The classifications derived from permuted data were compared to the actual clinical diagnostic status over 1000 permutations, and the reported *p*-value reflects the proportion of these permutations that resulted in accuracy that was better for the permuted labels than the actual labels.

External validity of the machine classifier measure was assessed by correlating the machine-derived classifier scores with clinician-derived ADOS-2 (Social Communication) scores from the same participants. As a final control procedure, to exclude the possibility that any patterns of results were driven by systematic differences in experimenter behavior, another SVM classifier was trained on language produced by the experimenter during the same conversations with identical analysis pipeline and validation procedure applied. For an analysis of which LIWC categories most strongly drove the classification, see Fig. S3. As previously reported, gross measures of linguistic behavior of the autism and typical groups were similar: there were no statistical differences in the proportion of time that participants (vs. the experimenter) spent speaking [t(37) = 0.31, *p* = 0.76], the total number of words produced [t(37) = –1.4, *p* = 0.17] the count of speaking turns [t(37) = 0.46, *p* = 0.65], or the number of words per sentence [t(37) = 0.01, *p* = 0.99].

### MRI imaging and ROI selection

EPIs and a T1 image were acquired on a GE HDxt 3 T scanner. The functional scan TR was 2 s, 64 × 64 matrix, sagittal acquisition to reduce effects of speech articulator movement. Pre-processing was performed with AFNI. Extreme values were attenuated with *3dDespike*, then volumes were slice-time corrected and co-registered to the T1 scan. Motion and physiological noise were reduced by regressing out cardiac and respiratory movements (RETROICOR), along with timeseries for white matter, ventricles, and the first 3 principal components of a combined white-matter and ventricle mask (*aCompCorr*). Full details are in Jasmin et al., 2019. Having established a behavioral measure of typical language use, we then used a data-driven procedure to localize brain areas that showed the greatest functional connectivity in autism relative to the neurotypical control group. First, for each functional MRI conversation scan, a global connectivity map (or ‘whole-brain connectedness map’) was calculated. To do this, (1) each gray matter voxel was correlated with every other gray matter voxel, (2) those correlations were averaged together, and (3) the average correlation was stored back in the original voxel’s location^1,13,22,23^. Next, a contrast of Autism > Typical participants was performed on the connectedness maps after fitting a linear mixed effects model on the data with AFNI’s *3dLME*, controlling for Motion and Age (model = [Connectedness ~ Group + Age + Motion + (1 | Participant)].

**Figure 1:**
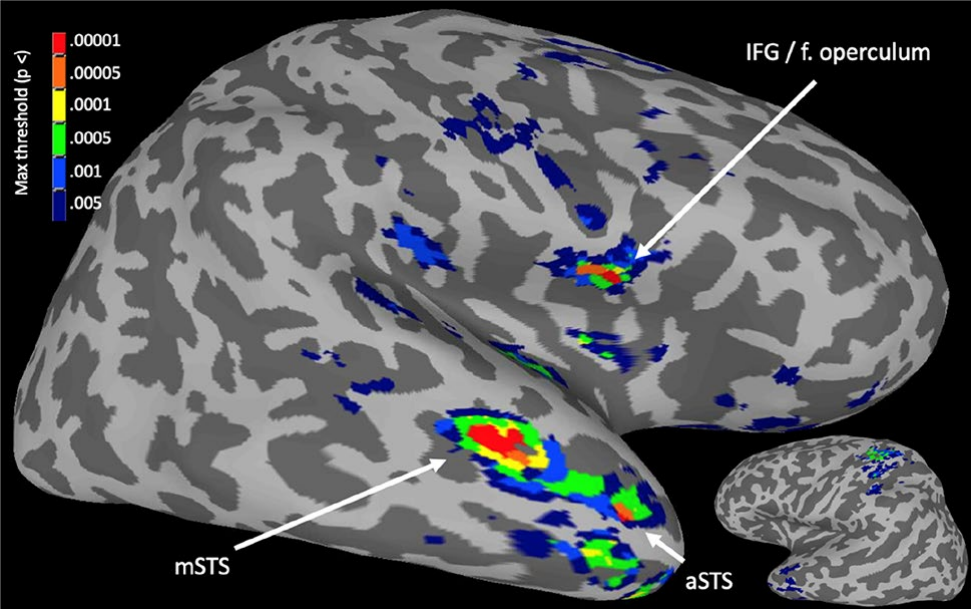
Right IFG, right mSTS, and right aSTS showed greatest autism > typical whole-brain connectedness across statistical thresholds. Inflated surfaces of the right hemisphere (large) and left hemisphere (small, inset). Colors indicate most stringent threshold survived.

Different combinations of voxel-wise significance thresholds and cluster extent thresholds result in different patterns of significant results. We sought to identify the brain areas most robust to choice of threshold by assessing voxel-wise and cluster-wise significance across a range of thresholds. The Autism > Typical contrast was thresholded at P < 0.005, 0.001, 0.0005, 0.0001, 0.00005, and 0.00001, with corresponding cluster extent thresholds of *k* ≥ 51, 22, 15, 7, 5, and 2 (determined using AFNI’s *3dClustSim* with empirically derived spatial autocorrelation function, -acf; see^24,25^). The significance maps from each thresholding procedure were binarized (1 if significant, 0 otherwise) and combined to produce a map that illustrated the maximum threshold survived by each voxel). Only three regions showed significantly greater whole-brain functional connectivity in autism at every threshold tested: right mid-superior temporal sulcus (mSTS), right anterior STS, and right IFG/operculum (Fig. 1; Table S1). These regions were selected for further analysis (at *P* < 0.0001, corrected, where each had 10 or more voxels, mitigating effects of between-subject anatomical variability).

For each of these three target ROIs, we assessed the correlation between whole-brain functional connectivity and Classifier Score by again fitting linear models with whole-brain connectedness (“Connectivity”) predicted by Classifier Score, Group (Autism or Typical) and their interaction. The interaction effect, critical for assessing compensation, was tested for each of the ROIs at a Bonferroni-corrected threshold of *P* < 0.05/3 tests = 0.0167.

### Ethical approval and consent to participate

The experiment was approved by the NIMH Institutional Review Board (protocol 10-M-0027, clinical trials number NCT01031407). Informed consent was obtained for all participants.

## Results

First, we assessed the accuracy and validity of the behavioral measure by evaluating how well the classifier could distinguish Autism and Typical participants when trained on participants’ speech. As a control, we also assessed whether the classifier could distinguish Autism and Typical participants if trained on the experimenter’s speech. External validity was assessed by comparing how the classifier scored autism participants during the interactions to how a clinician had scored the same participants using the ADOS-2. Finally, the relationship with functional connectivity was assessed. If elevated functional connectivity is compensatory, it should correlate with typical behavior for the Autism participants, but not for Typical participants (who should have no need to compensate).

### Classifier accuracy and validity

**Figure 2:**
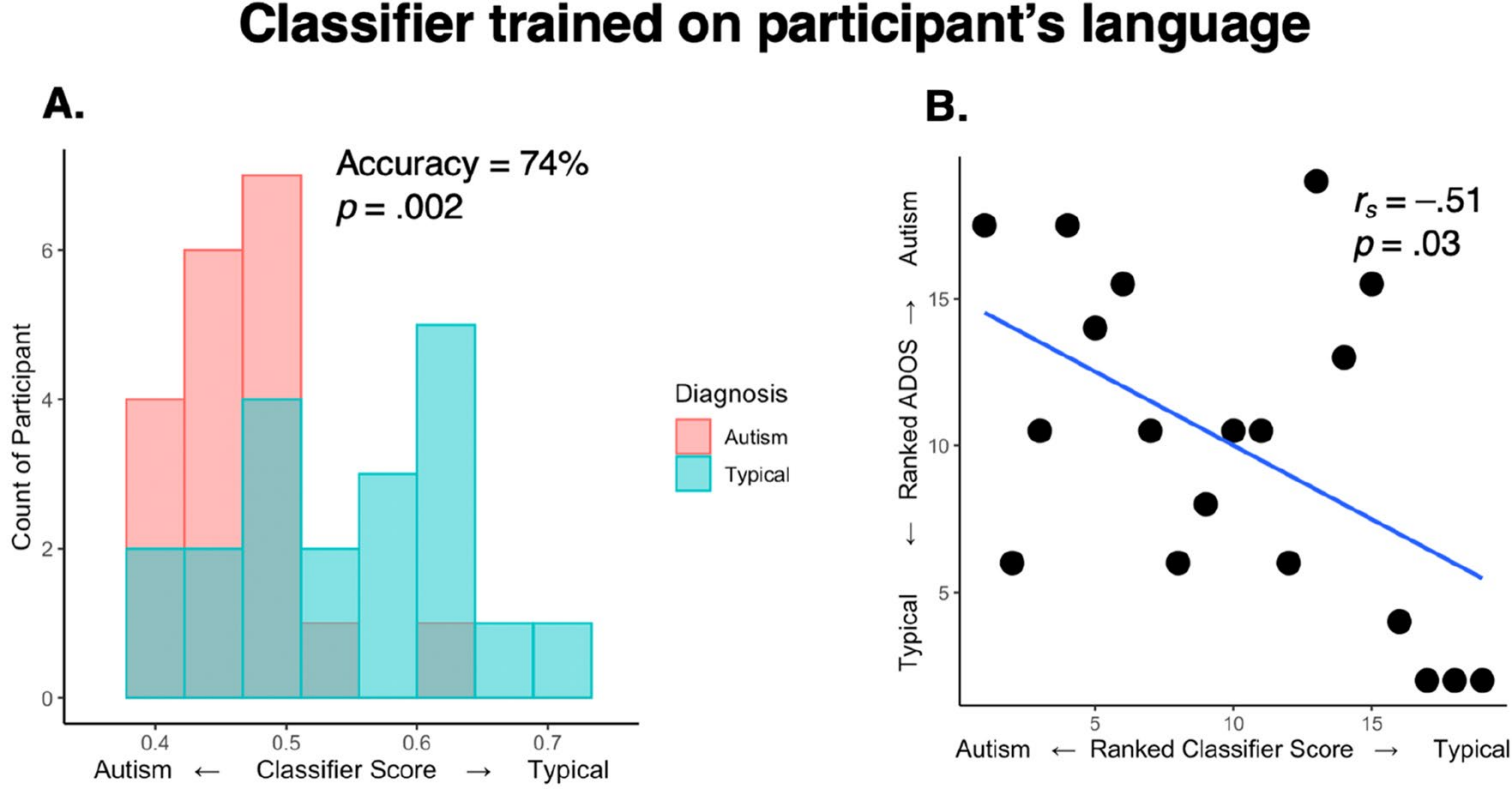
Classifier scores distinguished autism and typical participants and correlated with ADOS-2 social communication scores. **(A)** Histogram of mean classifier scores by diagnostic group and **(B)** ranked clinician ADOS-2 social communication subscores of autism symptom severity plotted by ranked classifier score.

The overall accuracy of the classifier trained on participant speech was 74% (*P* = 0.002 by permutation test; Fig. 2A). Autistic participants with more Typical classifier scores also displayed fewer autistic signs during their diagnostic observation performed a clinician (correlation with ADOS-2 Social Communication scores, Spearman *r* = –0.51, *P* = 0.03; Fig. 2B). It is of note that the two autism participants who were misclassified as typically developed by the classifier had the minimum ADOS-2 Social Communication scores required for diagnosis (i.e., a score of 7).

As a control, the same classification analyses were performed on the experimenter’s speech. Classifier accuracy dropped to 59%, which was statistically indistinguishable from chance (*p* = 0.13 by permutation test; Fig. S1). The scores derived from the experimenter’s speech furthermore did not correlate with ADOS-2 scores (Spearman *r* = –0.20, *P* = 0.42; Fig. S1). ROC curve analyses also indicated good classification when using participant speech, but poor classification when using experimenter speech (Fig. S2).

### Typical language behavior is associated with elevated functional connectivity

**Figure 3:**
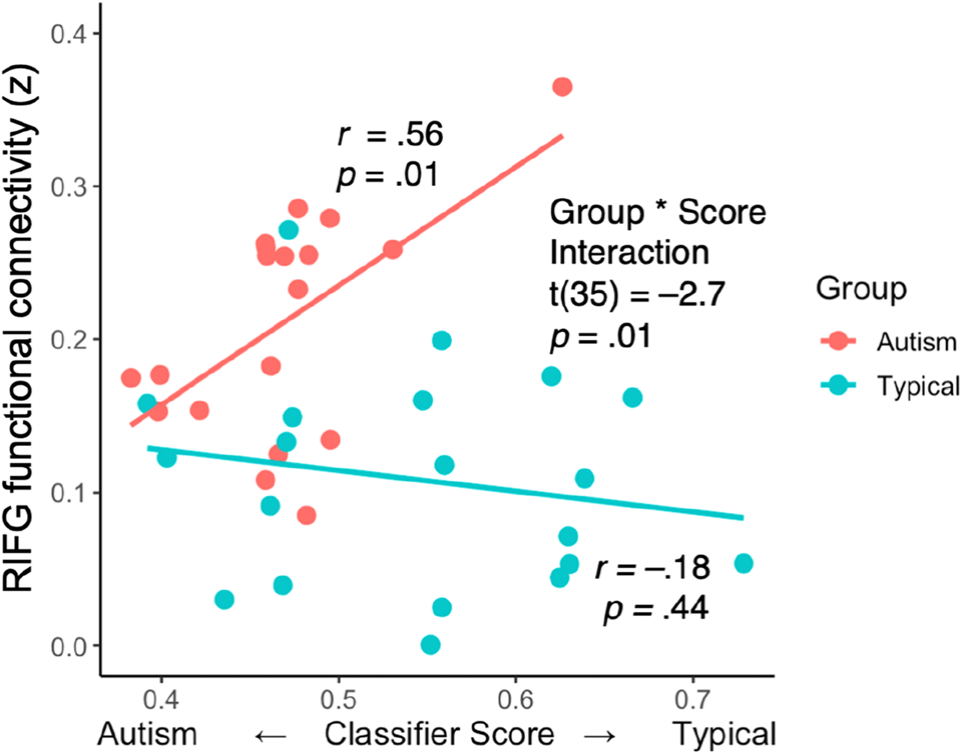
Typical language production was correlated with higher RIFG connectivity in autism participants. Scatter plot of whole-brain connectivity of RIFG plotted against classifier score for the autism (red) and typical (teal) groups.

Having established the validity of the linguistic measure, we examined its relationship with functional connectivity during the sessions. Three regions of interest were examined: mSTS, aSTS and RIFG. For each of these, the interaction of Group with Classifier Score was tested. Only the test for RIFG survived Bonferroni correction. (Right mSTS survived an uncorrected threshold of P < 0.05; for transparency we report this analysis in the Supplement (Fig. S4), and the pattern was qualitatively similar to that found in the RIFG). For RIFG, the slope of the correlation between functional connectivity and classifier score differed between the two groups (interaction *t*(35) = –2.7, *p* = 0.01; Fig. 3): in the Autism group, more typical classifier scores were associated with higher functional connectivity (*r*(17) = 0.56, *p* = 0.01), but this pattern failed to be detected in the Typical group (*r*(17) = –0.18, *p* = 0.44; Fig. 3). The Supplement contains further analyses establishing that the results are not attributable to global factors such as head motion, respiration, or arousal levels (Fig. S5) and that the linear model is robust (Fig. S6).

**Figure 4:**
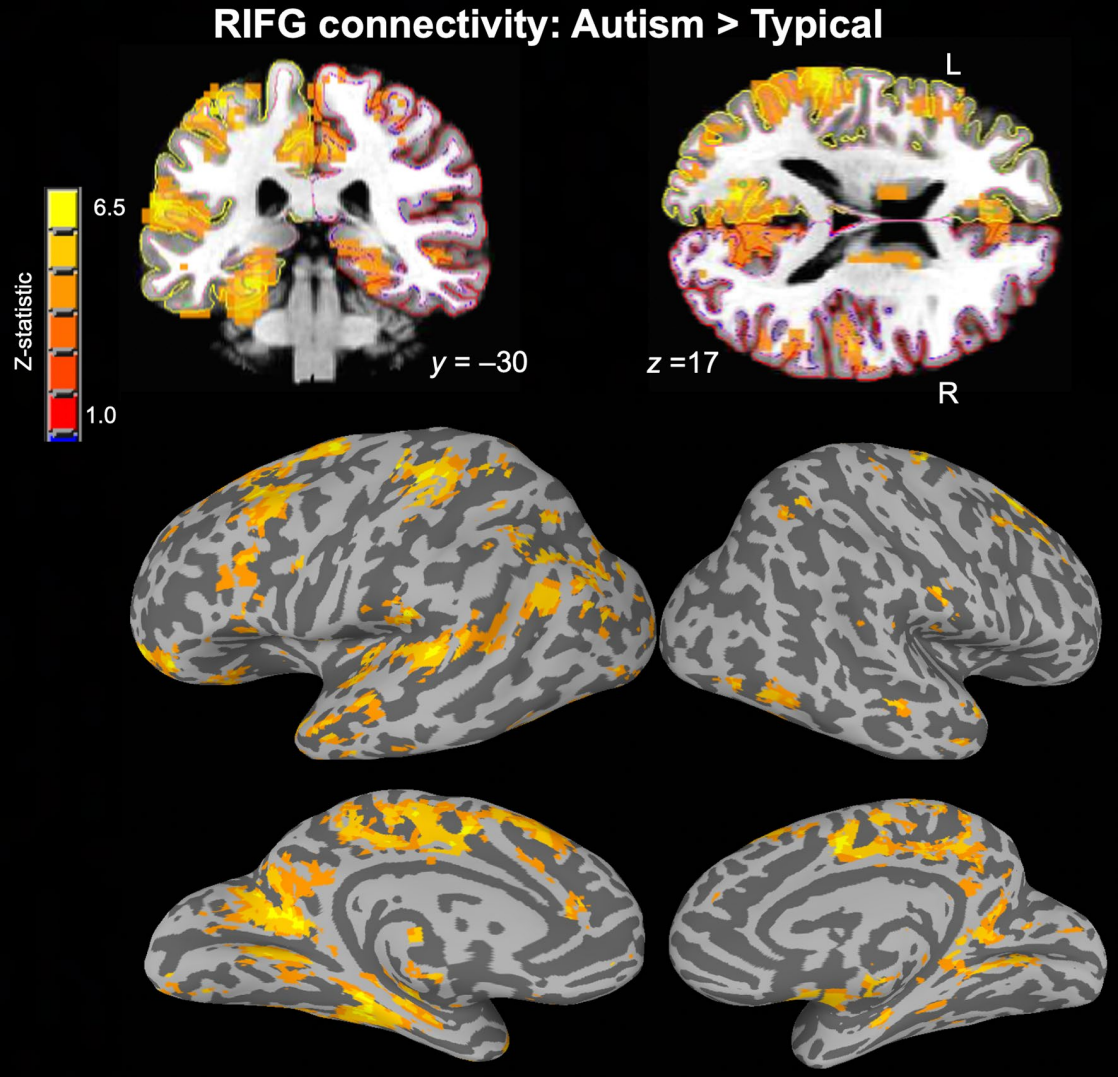
Compensation takes place through cross-hemispheric connectivity between left hemisphere social communication areas and right IFG. Coronal and axial sections, and inflated surfaces showing clusters with significant autism > typical functional connectivity with RIFG during conversation (P < .001, corrected). More than twice as many significant voxels were in the left hemisphere, and the strongest results were in regions involved with social communication and language.

Our neural measure, average “connectedness” with the whole brain, gives a broad indication of the level of functional involvement of a given region (in this case RIFG). However, the specific pattern of connectivity with the RIFG is unclear without follow-up analyses. We therefore ran an exploratory seed-based functional connectivity analysis using the RIFG to determine which other regions were involved in overconnectivity. A group comparison (Autism > Control, P < 0.001, corrected) was performed after fitting a linear mixed effects model to the data with *3dLME* [Connectedness ~ Group + Age + Motion + (1 | Participant)]. The results indicated that more than twice as many left-hemisphere voxels were over-connected with RIFG (5317 voxels) compared to right-hemisphere voxels (2452 voxels). The most prominent results were in left hemisphere areas associated with language and social interaction, such as left inferior frontal gyrus, left superior temporal gyrus, temporal pole, and left temporo-parietal junction (Fig. 4). The compensatory effect of right IFG connectivity appears to be driven strongly by its coordination with contralateral left hemisphere language areas.

## Discussion

In a previous study we detected widespread functional connectivity increases in autism during conversation. Here we found that autism participants with higher functional connectivity, particularly involving the RIFG, produced more typical language during conversations. This result helps to clarify the relationship between functional connectivity and performance of a naturalistic social task in autism, namely that functional connectivity increases are in some cases compensatory—helping rather than hindering social communication.

Why did RIFG emerge as a particularly important region for compensation? Conversation of course relies heavily on speech and language production, and our behavioral measure was derived entirely from spoken language. Although language is typically left-lateralized, there is evidence that the contralateral right hemisphere homologs of left-hemisphere language areas are recruited when task demands are high, both in typically-developed individuals and patient groups (so called ‘spillover’ processing^26^). Relating to autism and RIFG specifically, activity in RIFG (the homolog of Broca’s area) has been shown to increase when autism participants integrate language with social information such as age and gender^27^. Under this ‘spill-over’ account, the reason RIFG is heavily involved is that conversation is difficult for autism participants, and contralateral LIFG cannot meet task demands on its own. Our results are also consistent with a recent paper by Persichetti and colleagues, who found that the relative lack of cross-hemisphere interactions from right-hemisphere language homolog regions in autism was associated with poorer verbal ability^28^. A second and not-necessarily mutually exclusive possibility is that RIFG is playing a role in ‘executive function’, a process that has been proposed to assist compensation in developmental disorders^10^.

A minor result from Jasmin et al., 2019, was that RIFG connectivity with R parahippocampal gyrus was positively correlated with SRS scores, suggesting that functional connectivity increases correlate with more severe core autism deficits. That result is not necessarily inconsistent with the findings reported here. SRS and ADOS-2 scores are only weakly correlated^29,30^, which suggests they may measure at least partly different aspects of the condition.

### Limitations

The primary limitation of this study is the sample size. Although a very large amount of data was collected on each participant, the sample size in terms of number of participants is on the low side of current studies involving group comparisons in ASD. Future work should endeavor to replicate this finding in much larger samples. It would also be helpful to have a replication sample in the design, so that any discoveries could be confirmed within the same experiment.

The results do not fully address the question of which patterns of connectivity reflect underlying deficits and which may have developed to compensate for deficits. Further work should clarify this by quantifying other aspects of behavior and relating this to ongoing neural connectivity. The present study only used counts of spoken words in various categories as raw data. Other studies could examine, for example, acoustic aspects of vocal production such as prosody, eye movements, or hand gestures. Still other studies might address more subtle communication abilities such as how theory of mind problems are solved.

Further work should investigate a more diverse set of participants. Participants in this study had normal to relatively high IQ. It remains to be seen whether individuals with lower IQs also compensate, and if they do, whether they use a similar or different neural strategy. It has been suggested that females with autism may compensate more successfully than males^31^. Future studies could concentrate on other populations such as female participants, or perhaps so-called ‘unaffected’ siblings of individuals with autism, who may exhibit only mild autistic traits and receive no diagnosis due to a covert neural compensation strategy^10^.

In this study, the experimenter was neurotypical, making it difficult to distinguish whether the participant's autism status or the match/mismatch in diagnosis affected the results. Behavioral research has shown that people tend to have better rapport when they have similar neurotypes^32^. Future neural studies should investigate the relationship between language and compensatory functional connectivity in dyads where both partners have autism.

Finally, the classifier training method we used treated all observations (the 115 conversations) as independent, although in actuality these observations were contributed by only 39 participants. We made this choice to maximize the size of the training set and prioritize measurement stability. Future work, in larger samples of participants, could opt for more sophisticated models that take into account non-independence of observations.

## Conclusions

The findings suggest that right inferior frontal functional connectivity increases in autism during spontaneous social communication reflect a neural compensation strategy. The classifier-based behavioral method described here has wide applicability for studying compensation in developmental disorders. A main benefit is that it is not necessary to have an a priori definition of typical behavior. Behavior of any two groups of participants who are matched for relevant variables but differ categorically in diagnostic category could be submitted to a classification algorithm, and the resulting probability score could be used as an index of behavioral ‘performance’ and linked with neural measures.

## Supplementary Information

Supplementary Information.

## Data Availability

The datasets used and/or analysed during the current study are available from the corresponding author on request.
